# Advances in the Field of Cutaneous Malignancies: Background and Clinical Management

**DOI:** 10.3390/jcm15176751

**Published:** 2026-08-31

**Authors:** Péter Holló, Zsuzsanna Lengyel, András Bánvölgyi, Norbert Kiss

**Affiliations:** 1 Department of Dermatology, Venereology and Dermatooncology, Semmelweis University, 1085 Budapest, Hungary; 2 Department of Dermatology, Venerology and Oncodermatology, Medical School, Clinical Center, University of Pécs, 7632 Pécs, Hungary

Cutaneous malignancies constitute a growing clinical and public health burden, encompassing common keratinocyte carcinomas, melanomas, and a heterogeneous group of rare neoplasms. Their management increasingly depends not only on early recognition but also on precise characterization, appropriate staging, and individualized treatment selection [1,2,3,4,5,6]. Recent advances in non-invasive imaging, teledermatology, and artificial intelligence (AI) are expanding the information available before biopsy or treatment and are helping integrate diagnosis with therapeutic planning [6,7,8,9,10].

This Special Issue, “Advances in the Field of Cutaneous Malignancies: Background and Clinical Management”, brings together five contributions that reflect this evolution across diagnosis, characterization, and treatment. The included articles address periocular tumor imaging, teledermatology-based melanoma referral, a rare aggressive cutaneous lymphoma, field-directed therapy for actinic keratoses, and sentinel lymph node biopsy in head and neck melanoma.

Earlier and more accurate diagnosis remains a central objective in dermato-oncology. Chello et al. review reflectance confocal microscopy, optical coherence tomography, and line-field confocal optical coherence tomography for periocular skin tumors [11]. In this anatomically sensitive region, non-invasive imaging may increase diagnostic confidence, provide complementary information on lesion architecture, and support tissue-sparing management. However, the authors appropriately emphasize that the available evidence remains limited and that these modalities should complement rather than replace histopathology [11,12,13,14,15,16,17,18,19].

These observations are consistent with broader evidence showing that multimodal assessment may be more informative than reliance on a single technique [7,20,21,22]. Similarly, non-invasive imaging may increasingly assist basal cell carcinoma subtyping, which is clinically relevant because subtype influences treatment selection and recurrence risk [23,24,25].

Access to specialist assessment is another determinant of timely diagnosis. Cebolla-Verdugo et al. compared melanomas diagnosed through conventional face-to-face referral with those identified through a teledermatology-based pathway [26]. The teledermatology cohort showed a more favorable clinicopathological profile, including lower Breslow thickness, although the observational design and comparison of different time periods preclude causal conclusions [26]. Their findings complement the meta-analysis by Martyin et al., which found high diagnostic concordance for teledermatology and showed that adding dermoscopy significantly improved concordance for skin cancer [27]. Together, these studies support the structured integration of teledermatology into referral pathways while emphasizing the importance of image quality, clinical information, and appropriate escalation to in-person assessment.

Technological progress does not diminish the importance of clinicopathological expertise. Jaramillo et al. describe primary cutaneous CD8-positive aggressive epidermotropic cytotoxic T-cell lymphoma, a rare neoplasm characterized by rapid progression and limited therapeutic options [28]. The case illustrates how diagnostic delay may arise when uncommon malignancies mimic infectious or inflammatory disorders. It also underscores the continuing need for careful clinical correlation, histopathology, immunophenotyping, and multidisciplinary evaluation [28,29].

Cantisani et al. report their experience with topical 4% 5-fluorouracil in patients with actinic keratoses, observing high clearance and acceptable tolerability [30]. These findings reinforce the established role of 5-fluorouracil as a field-directed treatment, particularly in patients with extensive actinic damage [4,31,32].

Lázár et al. evaluate sentinel lymph node biopsy for head and neck melanoma in a medium-volume regional center and report complete sentinel-node identification without major complications [33]. Their study supports the feasibility of multidisciplinary staging outside high-volume centers and highlights the contemporary role of sentinel-node status in prognosis and selection for adjuvant therapy [1,34,35,36,37].

Collectively, the articles in this Special Issue demonstrate that progress in cutaneous oncology depends on combining innovation with clinical judgment. Non-invasive imaging, teledermatology, pathology, field therapy, and surgical staging address different points along the same patient pathway. AI may further connect these domains through multimodal analysis and decision support, but prospective validation, standardized protocols, transparency, and equitable implementation remain essential [8,38,39,40,41,42]. We thank the authors for their valuable contributions and the reviewers for their careful evaluations. We hope that this collection will encourage further multidisciplinary research and support increasingly precise, accessible, and patient-centered care for cutaneous malignancies.

## References

[1] Garbe C., Amaral T., Peris K., Hauschild A., Arenberger P., Basset-Seguin N., Bastholt L., Bataille V., Brochez L., Del Marmol V. (2025). European consensus-based interdisciplinary guideline for melanoma. Part 2: Treatment—Update 2024. Eur. J. Cancer.

[2] Peris K., Fargnoli M.C., Garbe C., Kaufmann R., Bastholt L., Seguin N.B., Bataille V., Marmol V.D., Dummer R., Harwood C.A. (2019). Diagnosis and treatment of basal cell carcinoma: European consensus-based interdisciplinary guidelines. Eur. J. Cancer.

[3] Stratigos A.J., Garbe C., Dessinioti C., Lebbe C., van Akkooi A., Bataille V., Bastholt L., Dreno B., Dummer R., Fargnoli M.C. (2023). European consensus-based interdisciplinary guideline for invasive cutaneous squamous cell carcinoma: Part 2. Treatment-Update 2023. Eur. J. Cancer.

[4] Kandolf L., Peris K., Malvehy J., Mosterd K., Heppt M.V., Fargnoli M.C., Berking C., Arenberger P., Bylaite-Bučinskiene M., Del Marmol V. (2024). European consensus-based interdisciplinary guideline for diagnosis, treatment and prevention of actinic keratoses, epithelial UV-induced dysplasia and field cancerization on behalf of European Association of Dermato-Oncology, European Dermatology Forum, European Academy of Dermatology and Venereology and Union of Medical Specialists (Union Européenne des Médecins Spécialistes). J. Eur. Acad. Dermatol. Venereol..

[5] Dippel E., Assaf C., Becker J.C., von Bergwelt-Baildon M., Bernreiter S., Cozzio A., Eich H.T., Elsayad K., Follmann M., Grabbe S. (2022). S2k-Guidelines—Cutaneous lymphomas (ICD10 C82–C86): Update 2021. J. Dtsch. Dermatol. Ges..

[6] Rosenthal A., Juhasz M., Zhang J., Ticknor I., Gharavi N., Man J. (2024). Survival outcomes of rare cutaneous malignancies within an insured cohort of patients, 1988–2018. J. Am. Acad. Dermatol..

[7] Varga N.N., Gulyás L., Meznerics F.A., Barkovskij-Jakobsen K.S., Szabó B., Hegyi P., Bánvölgyi A., Medvecz M., Kiss N. (2025). Diagnostic Accuracy of Novel Optical Imaging Techniques for Melanoma Detection: A Systematic Review and Meta-Analysis. Int. J. Dermatol..

[8] Szondy I., Martyin K., Mohos G., Kiss T., Szabó K., Boostani M., Goldust M., Grzybowski A., Bánvölgyi A., Kiss N. (2026). Artificial Intelligence in skin disease therapeutics: From drug discovery to personalized treatment pathways. Presse Med..

[9] Boostani M., Siegel A.P., Eathara A., Kiss N., Paragh G., Tsoukas M.M., Avanaki K. (2026). Skin imaging techniques for melanoma detection: A scoping review of modalities, clinical viability, and deployment strategies. J. Investig. Dermatol..

[10] Boostani M., Lallas A., Goldust M., Nádudvari N., Lőrincz K., Bánvölgyi A., Holló P., Wikonkál N.M., Paragh G., Kiss N. (2025). Diagnostic performance of multimodal large language models in distinguishing melanoma from nevi in clinical and dermoscopic images. JAAD Int..

[11] Chello C., Gemma G.P.A., Sadun R., Ambrosio L., Campanale E.A., Cappilli S., Pellacani G. (2026). In Vivo Non-Invasive High-Resolution Imaging for the Evaluation of the Periocular Skin Area: A Comprehensive Review of the Literature. J. Clin. Med..

[12] Babaee E., Boostani M., Charmi M., Avanaki K. (2026). Robust image quality evaluation in optical coherence tomography of skin using global, region-independent metrics. Sci. Rep..

[13] Shahriari N., Grant-Kels J.M., Rabinovitz H., Oliviero M., Scope A. (2021). Reflectance confocal microscopy: Diagnostic criteria of common benign and malignant neoplasms, dermoscopic and histopathologic correlates of key confocal criteria, and diagnostic algorithms. J. Am. Acad. Dermatol..

[14] Dinnes J., Deeks J.J., Saleh D., Chuchu N., Bayliss S.E., Patel L., Davenport C., Takwoingi Y., Godfrey K., Matin R.N. (2018). Reflectance confocal microscopy for diagnosing cutaneous melanoma in adults. Cochrane Database Syst. Rev..

[15] Ferrante di Ruffano L., Dinnes J., Deeks J.J., Chuchu N., Bayliss S.E., Davenport C., Takwoingi Y., Godfrey K., O’Sullivan C., Matin R.N. (2018). Optical coherence tomography for diagnosing skin cancer in adults. Cochrane Database Syst. Rev..

[16] Bergeron S., Arthurs B., Sanft D.M., Mastromonaco C., Burnier M.N. (2021). Optical Coherence Tomography of Peri-Ocular Skin Cancers: An Optical Biopsy. Ocul. Oncol. Pathol..

[17] Razi S., Kuo Y.H., Pathak G., Agarwal P., Horgan A., Parikh P., Deshmukh F., Rao B.K. (2024). Line-Field Confocal Optical Coherence Tomography for the Diagnosis of Skin Tumors: A Systematic Review and Meta-Analysis. Diagnostics.

[18] Cinotti E., Brunetti T., Cartocci A., Tognetti L., Suppa M., Malvehy J., Perez-Anker J., Puig S., Perrot J.L., Rubegni P. (2023). Diagnostic Accuracy of Line-Field Confocal Optical Coherence Tomography for the Diagnosis of Skin Carcinomas. Diagnostics.

[19] Donelli C., Suppa M., Tognetti L., Perrot J.L., Calabrese L., Pérez-Anker J., Malvehy J., Rubegni P., Cinotti E. (2023). Line-Field Confocal Optical Coherence Tomography for the Diagnosis of Skin Carcinomas: Real-Life Data over Three Years. Curr. Oncol..

[20] Abdalla B.M.Z., Reiter O., Godwin K., Gallagher R., Monnier J., Dos Reis Zuniga R.D., Jain M. (2025). Noninvasive Multimodal Imaging and Its Role in Diagnosing Skin Lesions in Dermatology: A Systematic Review and Meta-Analysis. Am. J. Clin. Dermatol..

[21] Deng J.-Y., Wei J.-P., Maimaitiyili N., Li X.-Y., Zheng Q.-T. (2026). Multimodal Non-Invasive Skin Imaging in Dermatology: Current Modalities, Clinical Applications, and Future Integration. Diagnostics.

[22] Horton L., Fakhoury J.W., Manwar R., Rajabi-Estarabadi A., Turk D., O’Leary S., Fotouhi A., Daveluy S., Jain M., Nouri K. (2025). Review of Non-Invasive Imaging Technologies for Cutaneous Melanoma. Biosensors.

[23] Boostani M., Pellacani G., Wortsman X., Suppa M., Goldust M., Cantisani C., Pietkiewicz P., Lőrincz K., Bánvölgyi A., Wikonkál N.M. (2025). FDA and EMA-approved noninvasive imaging techniques for basal cell carcinoma subtyping: A systematic review. JAAD Int..

[24] Guida S., Spadafora M., Ciardo S., Mirra M., Magi S., Mazzoni L., Kaleci S., Farnetani F., Rongioletti F., Stanganelli I. (2026). Improving basal cell carcinoma subtype diagnosis with dermoscopy and reflectance confocal microscopy: A multicentre prospective study. Clin. Exp. Dermatol..

[25] Longo C., Lallas A., Kyrgidis A., Rabinovitz H., Moscarella E., Ciardo S., Zalaudek I., Oliviero M., Losi A., Gonzalez S. (2014). Classifying distinct basal cell carcinoma subtype by means of dermatoscopy and reflectance confocal microscopy. J. Am. Acad. Dermatol..

[26] Cebolla-Verdugo M., Husein El-Ahmed H., Ramos-Pleguezuelos F.M., Ruiz-Villaverde R. (2025). Impact of a Teledermatology-Based Referral Model on Melanoma Diagnostic Pathways and Clinicopathologic Features: A Retrospective Comparative Study Between Face-to-Face Consultation (2019) and Teledermatology (2022) in a Tertiary Hospital. J. Clin. Med..

[27] Martyin K., Meznerics F.A., Bokor L.A., Szabó B., Hegyi P., Kiss N., Bánvölgyi A. (2026). Diagnostic accuracy of teledermatology for skin diseases: A systematic review and meta-analysis. Front. Med..

[28] Jaramillo J., Méndez-Flores K., Lascano N., Palacios-Álvarez S., Arias-Intriago M., Izquierdo-Condoy J.S. (2025). Primary Cutaneous CD8+ Aggressive Epidermotropic Cytotoxic T-Cell Lymphoma: A Rare and Aggressive Case Report with Clinical and Pathological Insights. J. Clin. Med..

[29] Roccuzzo G., Macagno N., Giordano S., Fava P., Quaglino P. (2025). New and emerging therapies in cutaneous T-cell lymphoma. Dermatol. Rep..

[30] Cantisani C., Di Guardo A., Paolino G., Balázs N., Boostani M., Kiss N., Conforti C., Feresin F., Carugno A., Gargano L. (2026). Efficacy of 4% 5-Fluorouracil Cream in the Treatment of Actinic Keratoses: A Single-Center Experience. J. Clin. Med..

[31] Toffoli L., Dianzani C., Bonin S., Guarneri C., Guarneri F., Giuffrida R., Zalaudek I., Conforti C. (2023). Actinic Keratoses: A Prospective Pilot Study on a Novel Formulation of 4% 5-Fluorouracil Cream and a Review of Other Current Topical Treatment Options. Cancers.

[32] Goon P.K., Clegg R., Yong A.S., Lee A.S., Lee K.Y., Levell N.J., Tan E.K., Shah S.N. (2015). 5-Fluorouracil “Chemowraps” in the Treatment of Multiple Actinic Keratoses: A Norwich Experience. Dermatol. Ther..

[33] Lázár P., Boa K., Mezőlaki N., Varga Z., Besenyi Z., Varga E., Németh I.B., Baltás E., Oláh J., Kis E.G. (2026). Sentinel Node Biopsy for Head and Neck Melanoma: A 12-Year Experience from a Medium-Volume Regional Center. J. Clin. Med..

[34] Mansini A., Aarohi S., Della Mura M., Sorino J., Cazzato G., Giubellino A. (2025). Sentinel Lymph Node Biopsy in Melanoma: Overview and Updates. Int. J. Mol. Sci..

[35] Cheng T.W., Hartsough E., Giubellino A. (2023). Sentinel lymph node assessment in melanoma: Current state and future directions. Histopathology.

[36] Brănişteanu D.E., Cozmin M., Porumb-Andrese E., Brănişteanu D., Toader M.P., Iosep D., Sinigur D., Brănişteanu C.I., Brănişteanu G., Porumb V. (2022). Sentinel Lymph Node Biopsy in Cutaneous Melanoma, a Clinical Point of View. Medicina.

[37] De Ravin E., Suresh N., Romeo D., Lu J., Shah M., Karakousis G., Moreira A., Rajasekaran K. (2022). Clinical Practice Guidelines on Sentinel Lymph Node Biopsy for Melanoma: A Systematic Review and Quality Appraisal Using the AGREE II Instrument. Ann. Surg. Oncol..

[38] Severin S., Boostani M., Salloum L., Bozsanyi S., Islam Z., Huss W., Arbesman J., Goldenberg A., Paragh G. (2026). Evaluating Large Language Models for Melanocytic Lesion Pathology Report Interpretation. J. Cutan. Pathol..

[39] Kassir M., Goldust M. (2026). AI in clinical diagnostics in dermatology: Applications, validation, and real-world use cases. Presse Med..

[40] du Crest D., Madhumita M., Enbiale W., Ruiz Postigo J.A., Malvehy J., Wongvibulsin S., Gupta S., Kittler H., Skayem C., Mahto A. (2026). AI and Digital Tools in Dermatology: Addressing Access and Misinformation. JMIR Dermatol..

[41] Karampinis E., Zoumpourli C.-M., Lallas A., Apalla Z., Paoli J., Akay B.N., Navarette-Dechent C., Biswanath B., Enechukwu N., Chai P. (2026). Perspectives on Artificial Intelligence in Dermatology: An International Cross-Sectional Study. Medicina.

[42] Behara K., Bhero E., Agee J.T. (2024). AI in dermatology: A comprehensive review into skin cancer detection. PeerJ Comput. Sci..

